# Highly Sensitive Biosensor for the Detection of Cardiac Troponin I in Serum via Surface Plasmon Resonance on Polymeric Optical Fiber Functionalized with Castor Oil-Derived Molecularly Imprinted Nanoparticles

**DOI:** 10.3390/bios16010012

**Published:** 2025-12-23

**Authors:** Alice Marinangeli, Pinar Cakir Hatir, Mustafa Baris Yagci, Alessandra Maria Bossi

**Affiliations:** 1 Department of Biotechnology, University of Verona, Strada Le Grazie 15, 37134 Verona, Italy; 2 Department of Biomedical Engineering, Faculty of Engineering and Natural Sciences, İstinye University, Ayazağa Mah. Azerbaycan Cad. (Vadistanbul 4A Blok) Sariyer, İstanbul 34396, Türkiye; pinar.hatir@istinye.edu.tr; 3 Nanotechnology and Advanced Materials Research Center, Istinye University, Sariyer, Istanbul 34396, Türkiye; 4 Koç University Surface Science and Technology Center (KUYTAM), Koç University, Istanbul 34450, Türkiye; byagci@ku.edu.tr

**Keywords:** molecularly imprinted polymers, cardiac troponin I, myocardial infarction, biosensors, plant oil based functional monomer, castor oil monomer

## Abstract

In this work, we report the development of a highly sensitive optical sensor for the detection of cardiac troponin I (cTnI), a key biomarker for early-stage myocardial infarction diagnosis. The sensor combines castor oil-derived biomimetic receptors, called GreenNanoMIPs and prepared via the molecular imprinting technology using as a template an epitope of cTnI (i.e., the NR10 peptide), with a portable multimode plastic optical fiber surface plasmon resonance (POF-SPR) transducer. For sensing, gold SPR chips were functionalized with GreenNanoMIPs as proven by refractive index changes and confirmed by means of XPS. Binding experiments demonstrated the cTnI_nanoMIP-SPR sensor’s ability to detect both the NR10 peptide epitope and the full-length cTnI protein within minutes (t = 10 min), with high sensitivity and selectivity in buffer and serum matrices. The cTnI_nanoMIP-SPR showed an LOD of 3.53 × 10^−15^ M, with a linearity range of 1 pM–100 pM, outperforming previously reported sensor platforms and making it a promising tool for early-stage myocardial infarction detection.

## 1. Introduction

Cardiac troponin I (cTnI) is a specific biomarker for acute myocardial infarction (AMI) and is considered the gold standard in clinical practice for diagnosing myocardial injury [1]. Elevated levels of cTnI, namely ≥ 0.04 ng/mL [2], are released into the bloodstream following damage to myocardial cells, i.e., after 3–12 h from the reported condition [2], making its detection essential for the early diagnosis and proper management of AMI [3]. For this reason, the demand for accurate and reliable diagnostic technologies that are able to detect low concentrations of cTnI remains a critical challenge. Immunoassays, such as enzyme-linked immunosorbent assays (ELISAs) [4], chemiluminescent immunoassays (CLIAs) [5] and radioimmunoassays (RAIs) [6], are the most widely used methods for cTnI detection [7]. These methods provide high sensitivity and specificity due to the use of antibodies. However, they typically require a laboratory setting and specialized equipment, which may involve complex steps in preparation, and can be time-consuming and expensive. Furthermore, techniques such as RAI, which uses radioactive reagents, involve safety concerns and are subject to more stringent regulations [8]. To overcome the limitations of conventional assays, significant efforts have been focused on the development of alternative sensing platforms that are able to detect low levels of cTnI with high precision. In response to this challenge, over the last few years, sensors have emerged as promising alternatives to traditional methods, offering real-time detection and the potential for miniaturization and integration into portable devices [8]. Among these, sensors based on molecularly imprinted polymers (MIPs) have collected increasing attention due to their ability to offer selective and stable recognition of specific analytes [9,10,11]. MIPs are synthetic materials designed to have highly specific binding sites for a target analyte. These polymers are created through a template-assisted polymerization process, in which functional monomers and crosslinkers are polymerized in the presence of the target analyte, acting as a template [12,13]. After polymerization, the template is removed, leaving molecular cavities in the polymer that precisely match the size, shape, and functional groups of the original template. This process creates a polymer with specific recognition sites that are highly selective for the target molecule, much like antibodies, but with several key advantages. MIPs exhibit high chemical and thermal stability, a long shelf-life, and resistance to harsh environmental conditions. Unlike biological receptors, they can be produced with a simple process at a low cost and do not require cold-chain storage [14,15]. These features make MIPs particularly attractive for applications in point-of-care diagnostics, environmental monitoring, and clinical sensing platforms, where robustness, reproducibility, and scalability are essential. In this framework, advancements in MIP technology have led to improvements in the successful synthesis of polymers capable of recognizing macromolecules [16]. A particularly promising strategy has involved the use of epitope imprinting, in which short fragments of the target protein are used as a template for the imprinting process [17,18]. Peptides, which are smaller and more structurally undefined/flexible than full proteins, can serve as effective surrogates for the protein target. This strategy not only simplifies the synthesis process but also ensures that the MIP can recognize the protein in a highly specific manner by targeting key epitopes [19]. Several recent studies have demonstrated the feasibility of this method for cTnI recognition [20,21,22,23]. Building on these advances, we previously designed MIP receptors for cTnI, called GreenNanoMIPs, by adopting a peptide epitope imprinting strategy, selecting a specific fragment of the cTnI protein, the NR10 peptide, located in its C-terminal region [22]. This region was chosen due to its structural flexibility and lack of defined secondary structures, such as β-sheets, which facilitate both the imprinting process and subsequent molecular recognition [24,25]. Being located at the C-terminal region, it is accessible for recognition; it is among the targeted regions in ELISAs and was reported as an epitope for imprinting cTnI in the literature [20,21,22]. Moreover, the NR10 was selected because of its unique sequence (Peptide Atlas accession PAp00790335, 3601 experimental observations), which makes it highly suitable for the selective identification of human cTnI.

MIP-based sensors for cTnI detection, relying on electrochemical transduction, focused much of the innovation on improving the sensitivity of MIPs by integrating conductive nanomaterials to create more efficient and sensitive platforms. In particular, the MIP sensing layer was electrochemically polymerized, while doped with graphene and carbon nanotubes. Resulting in improved sensor sensitivity and signal amplification [26]. A notable example is Yola et al. [27], who developed an electrochemical sensor by electropolymerizing an MIP layer on a glassy carbon electrode (GCE) and enhancing the signal by doping it with boron nitride quantum dots. This approach resulted in a limit of detection (LOD) of 5 × 10^−4^ ng/mL, compatible with cTnI detection in complex biological matrices. In a similar vein, Ma et al. [28] layered graphene nanoplatelets and multi-walled carbon nanotubes on a GCE, followed by a chitosan deposition onto which a poly-methacrylic MIP layer was grown. This sandwich approach not only improved the electronic transmission rate but also enhanced the surface area, increasing the sensor’s sensitivity and improving the binding kinetics for cTnI detection, resulting in an LOD of 0.027 nM (i.e., 8 × 10^−4^ ng/mL). Hasabnis et al. [21] demonstrated the enhancement of MIP sensor performance by a strategy based on incorporating GQDs and AuNPs into the MIP layer. This combination allowed them to achieve a LOD of 0.5 pg/mL, showcasing signal amplification and increased sensitivity. Moreover, the GQDs and AuNPs incorporated into the MIP layer also improved the affinity, reproducibility, and specificity of the sensor, making it more suitable for translation to real-world applications. While nanomaterial-assisted electrochemical MIP sensors have greatly improved the analytical performance for cTnI detection, the integration of molecular imprinting with advanced optical techniques remains relatively under-explored. One notable exception is the work by Choudhary et al. [20], who developed a point-of-care surface plasmon resonance (SPR) sensor based on the Kretschmann configuration [29] and functionalized with epitope-imprinted nanoMIPs for the real-time detection of cTnI. Their system demonstrated high reproducibility and selectivity, with an LOD of 0.52 ng/mL and a dissociation constant of 2.99 × 10^−11^ M, confirming the superior affinity of the imprinted receptors. However, in this configuration the LOD still remained uncompetitive compared to clinical requirements, particularly those aimed at ultra-sensitive diagnostics (i.e., the level of cTnI in the bloodstream: 0.04 ng/mL to 1.4 ng/mL within 3–12 h) [2].

Besides conventional SPR platforms, D-shaped plastic optical fiber (POF) SPR represents a highly versatile and cost-effective transducer. The D-shaped POFs provide several advantages, including a high numerical aperture, large core diameter, and remarkable mechanical flexibility, which allows the fiber to withstand tighter bend radii than glass fibers. Additionally, it has easy manipulation and low cost. Furthermore, the D-shaped POF-SPR configuration supports efficient broadband light coupling and enables remote and real-time interrogations [30]. Thanks to these advantages, SPR-POF sensors have been successfully employed for a variety of analytical and clinical applications, including the detection of proteins, metabolites, and biomarkers, using antibodies, aptamers, or MIP recognition elements. It was demonstrated that the combination of nanoMIPs that have a hydrogel nature with the D-shaped POF-SPR can lead to significant enhancements in sensor sensitivity, achieving LODs for the detection of protein analytes in the low picomolar range [31]. In particular, the analyte binding event was shown to deform the MIP nanogels, resulting in the swelling of the nanoMIPs and, hence, in blue-shifts at binding [31], in accordance with earlier reports [32]. In the present work, we developed a portable D-shaped POF-SPR functionalized with GreenNanoMIP nanogels, customized for the recognition of cTnI, with the aim of achieving the sensitivity required for in serum cardiac failure marker detection within the diagnostic levels.

## 2. Materials and Methods

### 2.1. Materials

Acrylated methyl ricinoleate (AMR) was synthesized from castor oil as described in our previous work [33]. All solvents, Ethylene glycol methyl ether acrylate (EGMEA), Lithium phenyl-2,4,6-trimethylbenzoylphosphinate (LAP), *N*,*N*′-Methylenebis(acrylamide) (MBA), Human serum albumin (HSA), cytochrome C (Cyt C), Human serum Dimethyl sulfoxide (DMSO), Phosphate-Buffered Saline (PBS), Tween20, Tris-free base Buffer, 2-(*N*-morpholino)ethanesulfonic acid (MES), *N*-(3-Dimetilaminopropyl)-*N*’-ethylcarbodiimide (EDC), and *N*-Hydroxysuccinimide (NHS), were purchased from Sigma-Aldrich (Darmstadt, Germany). Cardiac troponin I (cTnI) was purchased from GenScript. The peptides NR10 of sequence NIDALSGMGR and NR11 of sequence NIDALSGMEGR were custom-synthesized from TAG-Copenhagen.

### 2.2. Synthesis of GreenNanoMIPs

GreenNanoMIPs were synthesized as reported in [22]. To summarize, AMR, EGMEA, and the template-peptide solution NR10 were combined and left for 15 min to form an intermolecular complex through noncovalent interactions. The appropriate solvent mixture (90:10, DMSO: H_2_O) was then added into a glass vial, maintaining a monomer concentration of 0.5% (wt/wt). MBA and the initiator (10% wt/wt relative to the total monomer weight) were introduced next. The vial was exposed to a 365 nm LED UV lamp for 2 min, after which the reaction was stopped. The solution was then treated with 2 mL of 50 mM Tris-free base and left in the dark for 30 min. The polymerization solution was transferred into dialysis membranes (12,000 Da MWCO) and dialyzed against 5 L of water, repeating this process three times. Finally, the solution was placed in a Falcon tube and freeze-dried. Non-imprinted polymer nanoparticles were produced using the same procedure but without the template-peptide.

### 2.3. Functionalization of SPR-Polymeric Optical Fiber (SPR-POF)

The gold surface of a D-shaped SPR-POF [30] was first plasma-cleaned and treated overnight at room temperature (RT) with α-lipoic acid at a concentration of 0.3 mM in 8% ethanolic solution, producing a self-assembled monolayer (SAM). Subsequently, the so-produced surface was activated with EDC/NHS in a two-step reaction. Firstly, 100 µL of EDC at a concentration of 20 mM in MES buffer (50 mM, pH 5.5) was added to the surface for 30 min at RT. Then, NHS at the final concentration of 20 mM in MES buffer (50 mM, pH 5.5) was mixed with GreenNanoMIPs (1 mg/mL) for 10 min at RT. Then, 100 µL of this solution was added to the surface and incubated for 2 h at room temperature. Lastly, Tris-free base was added to the surface for 10 min to block the EDC/NHS reaction [31]. At the end of the process, the SPR-POF was rinsed with MilliQ water and kept in MilliQ water ON at RT to remove all non-reacted elements. The functionalization process of the GreenNanoMIPs-SPR-POF was evaluated by monitoring the variations in the plasmonic wavelength at each step, which were computed in relation to the non-functionalized chip and considering MilliQ water as the surrounding solution.

### 2.4. X-Ray Photoelectron Spectroscopy (XPS)

XPS analyses of bare gold SPR-chip, α-lipoic acid-modified SPR-chip, and GreenNanoMIP-SPR-chip were carried out according to [34,35] by a Thermo Scientific K-Alpha XPS with Al K-alpha monochromatic radiation (1486.3 eV) using a 400 μm X-ray spot size. The pass energy was set to 50 eV, corresponding to an energy resolution of roughly 0.5 eV. The take-off angle was set to 90°. All measured peaks were deconvoluted and fit by using Avantage 5.9 software. C 1s peak at 284.5 eV was designated for the charge correction and peak assessment.

### 2.5. Portable Surface Plasmon Resonance (SPR)

Measurements were performed on a portable SPR Spectra340 (Moresense S.r.l., Milan, Italy) [36]. In particular, the SPR platform is based on a modified D-shaped SPR-POF chip (model RA1008 manufactured by Moresense S.r.l., Milano, Italy), connected to the equipment by a 3D-printed custom holder. More specifically, the experimental setup is based on a spectrometer with a VIS range of 500–730 nm and a white light source of 400–780 nm. The SPR spectra are elaborated by custom Software (Capture Spectrum Data ver.2.4.8), which mathematically analyzes the plasmonic track, identifying the minima by first and second derivatives.

### 2.6. Sensor Response to NR10 Peptide

The dose–response curves of the SPR sensor were obtained by testing the NR10 peptide concentrations ranging between 1 pM and 30 nM in PBS (10 mM, pH 7.4). The experimental measurements were carried out by placing 100 μL of the NR10 solution at different concentrations onto the SPR-POF chip. The SPR spectra were acquired every 3 min until 15 min incubation time. A washing step between each concentration was performed by gently washing the surface with PBS three times. The SPR spectra were obtained using a normalization process, which was carried out by dividing the transmitted spectra on a reference spectrum obtained in air. The absolute values of resonance wavelength variations (|∆*λ*|) at different target concentrations (*c*) were calculated with respect to the plasmonic spectra in the absence of the analyte as |∆*λ*| = *λ*_*c*_ − *λ*_0_, where *λ*_c_ corresponds to the resonance wavelength when the NR10 concentration is *c* and *λ*_0_ corresponds to the resonance wavelength of the blank (solution without the NR10). The experimental data obtained were fitted using the Hill equation model, which has been previously employed to describe binding behavior in nanoMIP-based plasmonic sensing systems [31], as reported in Equation (1):

(1) $$\lambda =\lambda_{\_end}\frac{c^{n}}{\left(K_{app}+c^{n}\right)}$$

where *λ* is the wavelength at concentration *c* of the ligand; *λ__end_* is the value at binding saturation; *n* is the Hill parameter, which correlates with the number of binding sites; *K_app_* is the apparent dissociation constant. The fitting was carried out by using OriginPro software (version 9.0, Origin Lab. Corp., Northampton, MA, USA).

### 2.7. Sensor Response to cTnI

The dose–response curves of the SPR sensor for the protein cTnI were obtained as described in the Section 2.6 paragraph. cTnI was tested at different concentrations ranging from 100 fM to 10 nM, and data were analyzed as described before.

### 2.8. Selectivity Test

To test the selectivity of GreenNanoMIPs grafted on SPR-POF, NR11 peptide (NIDALSGMEGR) was chosen as a competitor. The experimental measurements were carried out by placing 100 μL of the NR11 solution at different concentrations (from 1 pM to 10 nM) onto the SPR-POF chip. Additionally, the ability of GreenNanoMIPs grafted on SPR-POF to bind non-target proteins HSA and Cyt C was tested using different concentrations of the proteins (from 1 pM to 10 nM). The measurements and analysis were carried out as described in the Section 2.6.

### 2.9. Measurement of cTnI in Serum

SPR-POF chips modified with GreenNanoMIPs were tested with model serum and human serum, both spiked with a fixed concentration of cTnI (100 pM). In particular, the model serum was prepared with HSA at 1 mg/mL and then spiked with 100 pM of cTnI. Human serum from a pool of healthy donors was diluted 1:50 in PB 10 mM with the addition of 0.1% Tween 20. The experimental measurements were carried out by placing 100 μL of the serum spiked with a fixed concentration of cTnI onto the SPR-POF chip. The measurements and analysis were carried out as described in the Section 2.6.

## 3. Results

### 3.1. Preparation of the cTnI-NanoMIP-SPR-Sensor and Physical Characterization

**Figure 1:**
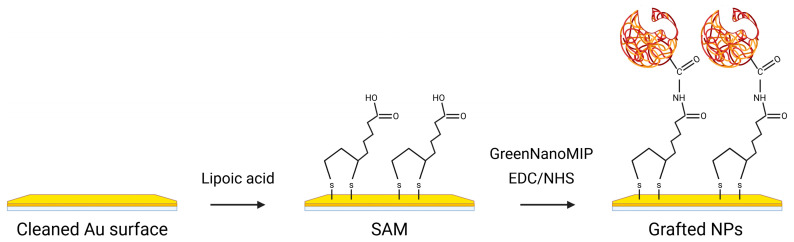
Schematic representation of the functionalization steps of the gold-coated POF surface with GreenNanoMIPs. The yellow surface represents the gold surface of the POF, while the red particles correspond to the GreenNanoMIPs.

The GreenNanoMIPs for the selective recognition of cTnI were prepared by us previously, starting from a castor-oil-based monomer, and the binding abilities of these GreenNanoMIPs were assessed with a time-resolved fluorescence spectroscopy, showing selectivity for the epitope template NR10 with an EC_50_ of 1.37 × 10^−11^ M and a stability in solution for over 1 year (details in Table S1 DLS, Figure S1 stability, Figure S6 SEM image) [22]. In the present work, we exploited the GreenNanoMIPs to devise a portable POF-SPR biosensor for the determination of cTnI. For this, the gold-coated POF surface was functionalized with the GreenNanoMIPs, as schematically illustrated in Figure 1, following the approach described by Cennamo et al. [31]. Initially, a self-assembled monolayer (SAM) was formed on the gold surfaces through the use of α-lipoic acid [37]. Subsequently, the carboxylic groups of the SAM were activated using a mixture of EDC and NHS to enable covalent coupling. The activated surface was then incubated with GreenNanoMIPs, which were immobilized through the formation of stable amidic covalent bonds. Finally, any unreacted active sites were blocked using Tris-free base buffer to prevent nonspecific adsorption [31].

**Figure 2:**
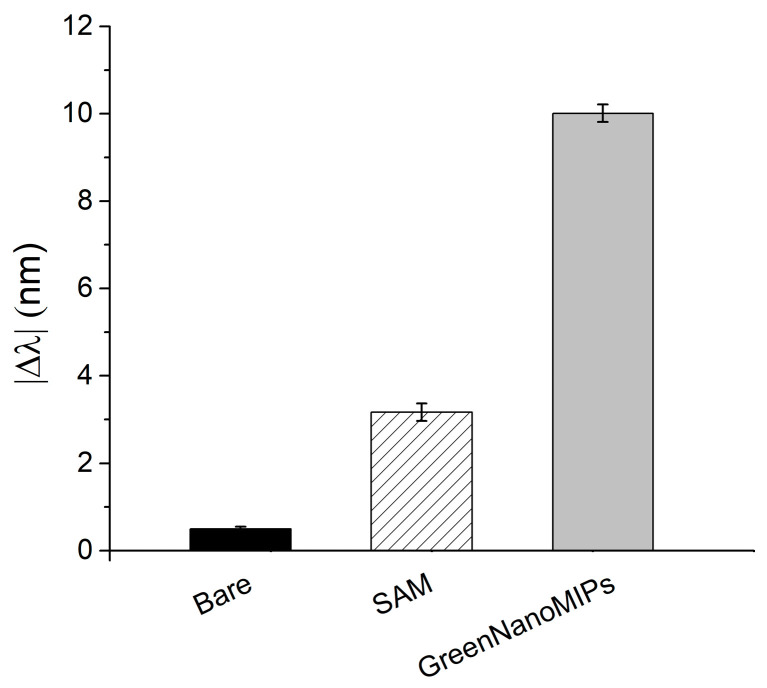
Absolute plasmonic resonance wavelength shifts (|Δ*λ*|) of the SPR-POF after each functionalization step for the immobilization of GreenNanoMIPs: solid black bar indicates bare gold; striped bar, SAM; gray bar, GreenNanoMIPs.

As reported in Figure 2, the effectiveness of the functionalization procedure was assessed by evaluating the plasmonic spectral changes as the |∆*λ*| computed in relation to that of the bare probe (i.e., cleaned, bare gold surface). Initially, the bare gold surface (*n* = 10) exhibited a plasmonic resonance at approximately 601.88 ± 1.44 nm. Following the formation of the α-lipoic acid SAM, a red-shift to 605.05 ± 1.77 nm was observed, indicating successful surface modification. Upon the grafting of GreenNanoMIPs, the resonance further shifted to 611.89 ± 1.27 nm, consistent with the increased thickness and the higher refractive index at the interface due to the presence of the polymeric layer. This functionalization procedure was repeated across multiple chips to evaluate reproducibility. As shown in Figure 2, the |Δ*λ*| observed for the formation of the α-lipoic acid SAM on the gold-coated POF was 3.17 ± 0.20 nm, and it further increased to 10.01 ± 0.20 nm after GreenNanoMIPs grafting. The standard deviations observed across multiple replicates (*n* = 10) were below ≤0.6%, confirming that the functionalization procedure yields consistently modified sensor surfaces with high reproducibility.

In support, X-ray photoelectron spectroscopy (XPS) analysis was carried out to independently confirm the functionalization of the gold-coated POFs with GreenNanoMIPs. XPS allowed us to inspect the surface chemical composition at each functionalization step (Table 1, details in Supplementary Information Figure S2) [34,35], verifying the presence of characteristic elemental signatures associated with the SAM and the polymeric GreenNanoMIPs layer, providing complementary evidence of successful surface modification. The chemical composition of the gold surface (C 9%; O 6%; N 0%) changed progressively with each modification step. The formation of the α-lipoic acid SAM was evidenced by an increase in carbon and oxygen contents (C 46.3%; O 25.6%, N 0%). The successful grafting of GreenNanoMIP was confirmed by the appearance of nitrogen and notable increases in both oxygen and carbon content (C 56%, O 23.5%, N 4.7%).

### 3.2. Kinetics of the Sensor Response

**Figure 3:**
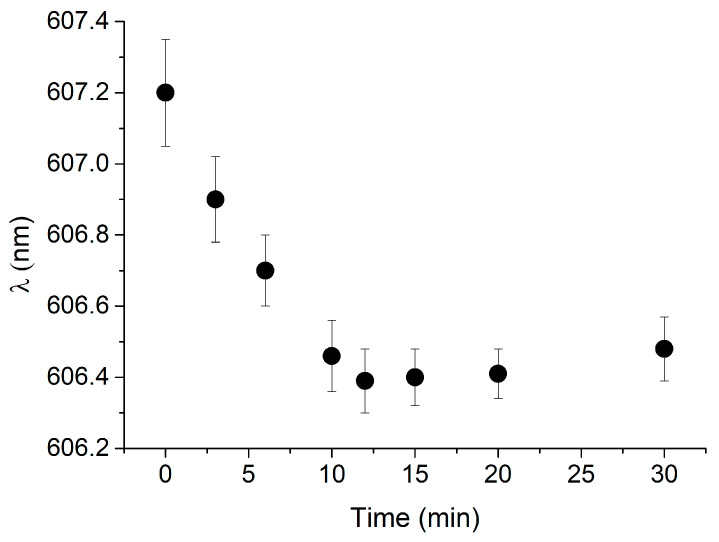
Kinetics of response of GreenNanoMIP-SPR-POF sensor: plasmonic resonance wavelength (*λ*) was plotted over time (min) for representative NR10 concentration (i.e., 100 pM).

The response time of the cTnI_nanoMIP-SPR-sensor was evaluated by monitoring the evolution of the plasmonic resonance wavelength (*λ*) over time during the binding process. Figure 3 reports the plasmonic wavelength shift (*λ*) recorded for a representative NR10 concentration, plotted as a function of time (minutes). The signal gradually stabilizes, reaching equilibrium after approximately 10 min of incubation. This stabilization indicates that the binding interaction between NR10 and the GreenNanoMIP layer achieves steady-state conditions within this time frame, confirming that reliable measurements can be obtained with a rapid total assay time of 10 min.

### 3.3. Sensor Response to NR10 Peptide and Selectivity

**Figure 4:**
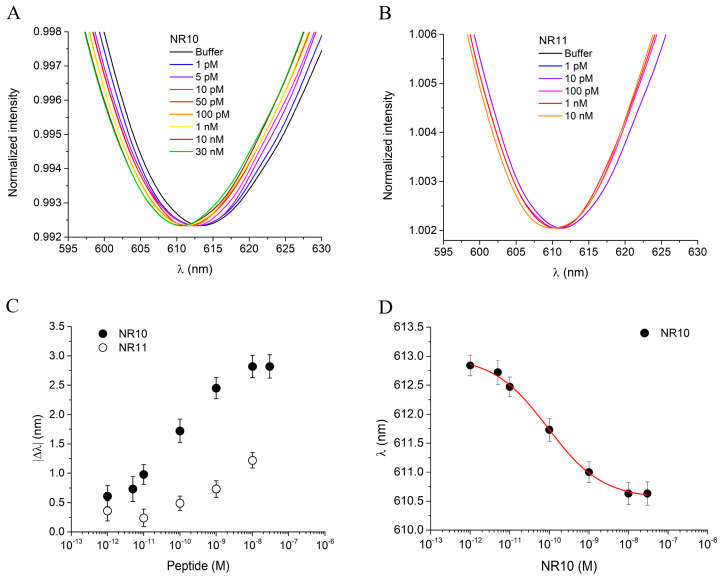
(**A**) Plasmonic spectra of GreenNanoMIPs-SPR-POF sensor incubated with increased concentrations of NR10 peptide (1 pM–30 nM). (**B**) Plasmonic spectra of GreenNanoMIPs-SPR-POF sensor incubated with increased concentrations of NR11 peptide (1 pM–10 nM). (**C**) Absolute plasmonic resonance wavelength shifts (|Δ*λ*|) as a function of peptide concentration for the templated peptide NR10 (black dots) and the non-templated peptide NR11 (open dots). (**D**) Binding curve of NR10, showing the plasmonic resonance wavelength (*λ*) as a function of NR10 peptide concentration, fitted with the Hill equation model (red line).

The binding performance of the cTnI_nanoMIP-SPR sensor was evaluated for the detection of the NR10 peptide in PBS, within a concentration range from 1 pM to 100 nM. Figure 4A shows the plasmonic spectra after 10 min of incubation for each NR10 concentration, following the measurement protocol described in the Section 2. A zoomed-in view of the resonance peaks for the NR10 binding is provided in the Supplementary Information (Figure S3A). A clear concentration-dependent blue-shift in the plasmonic resonance wavelength was observed, with a maximum |Δ*λ*| of approximately 3 nm, reflecting a variation in the local refractive index near the gold surface upon the binding of NR10 to the GreenNanoMIP layer. The occurrence of a blue-shift, rather than the red-shift typically associated with molecular adsorption on rigid plasmonic interfaces, has been explained by considering the soft hydrogel nature of the GreenNanoMIP layer [31,32]. The polymeric network of the GreenNanoMIPs can absorb water and vary its degree of hydration, undergoing swelling or shrinking depending on the surrounding environment, which affects the thickness of the sensing layer, thus changing the effective refractive index [32,38,39]. Upon binding with the hydrated target analyte, soft polymer matrices undergo local deformation, hydration, and structural rearrangement, yielding to the lowering of the refractive index, hence leading to a blue-shift of the signal [31,32].

To evaluate the selectivity of the cTnI_nanoMIP-SPR sensor, a structurally similar peptide, NR11, was used as a competitor, as shown in Figure 4B. A zoomed-in view of the resonance peaks for the NR11 binding is provided in the Supplementary Information (Figure S3B). NR11 differs from NR10 by a single additional glutamic acid (E) residue, resulting in comparable molecular weights (1033 Da for NR10 and 1162 Da for NR11), but with notable differences in physicochemical properties. Specifically, NR10 has an isoelectric point (pI) of 7.00, while NR11 has a lower pI of 4.07. The difference in pIs suggests that NR10 is more neutral at physiological pH (around pH 7.4), whereas NR11 is more acidic, potentially influencing its interaction with the MIP’s binding sites.

Additionally, the Grand Average of Hydropathy (GRAVY) scores were −0.11 for the NR10 peptide and −0.42 for the NR11 peptide. The GRAVY score reflects the hydrophobicity or hydrophilicity of a peptide, with negative values indicating a tendency towards hydrophilicity (water solubility) and positive values indicating hydrophobicity (tendency to avoid water). The more hydrophilic nature of NR11, as indicated by its more negative GRAVY score, suggests that NR11 may have different solubility and surface interaction properties compared to NR10, further influencing the binding selectivity of the sensor. When exposed to NR11 in concentrations ranging from 1 pM to 10 nM, no significant shift in the plasmonic resonance wavelength was observed (Figure 4B), highlighting the sensor’s high selectivity for the NR10 template. The dose–response curves, presented in Figure 4C, plot the absolute |Δ*λ*| as a function of analyte concentration on a semilogarithmic scale. The use of |Δ*λ*| values allows the normalization of results obtained from different sensing chips, ensuring data comparability and minimizing small baseline variations between experiments. Conversely, the fitting of the binding isotherm (Figure 4D) was performed using the absolute resonance wavelength (*λ*) rather than |Δ*λ*|, since this approach provides direct access to the parameters *λ__start_* and *λ__end_* of the fitting equation model, which are subsequently used to calculate the main operational parameters of the sensor. In particular, the curves for the templated peptide NR10 (black dots) and the non-templated peptide NR11 (open dots) exhibit distinct binding behavior, with NR10 producing a clear response and NR11 showing negligible interaction. The statistical variability of the measurements, calculated from three replicate tests on separate chips, was 0.45%, indicating very high reproducibility. Data obtained from the NR10 binding were fitted to the Hill equation model using OriginPro software (Figure 4D), and the related fitting parameters, along with the corresponding sensor parameters, are summarized in Table 2. In particular, the cTnI_nanoMIP-SPR-sensor exhibited a |Δ*λ*| of approximately 3 nm, with half-saturation corresponding to an apparent dissociation constant (*K_app_*) of 2.73 × 10^−11^ M. The statistical number of binding sites per particle, *n*, was found to be 0.49, suggesting that not all available binding sites were accessible for interaction. In terms of analytical performance, the cTnI_nanoMIP-SPR sensor exhibited a limit of detection (LOD) of 2.9 × 10^−14^ M, a limit of quantification (LOQ) of 9.83 × 10^−14^ M, and a sensitivity at low concentrations of 2.23 × 10^13^ M.

### 3.4. Sensor Response to cTnI and Selectivity

**Figure 5:**
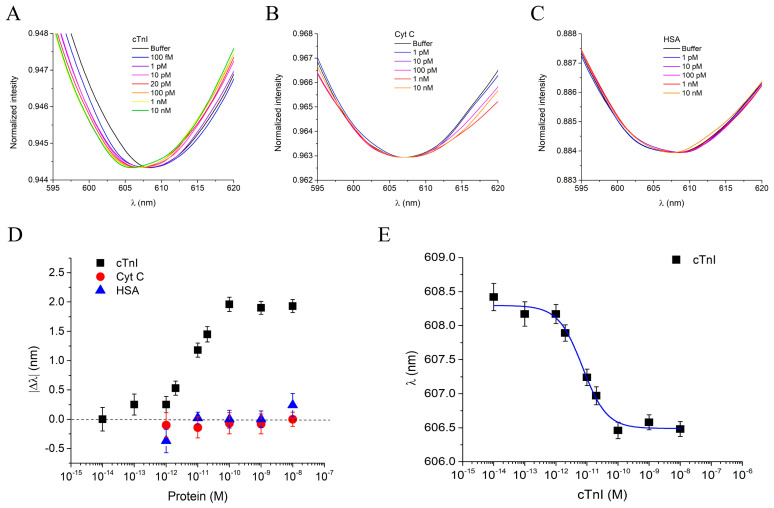
(**A**) Plasmonic spectra of GreenNanoMIPs-SPR-POF sensor incubated with increased concentrations of cTnI (100 fM–10 nM). (**B**) Plasmonic spectra of GreenNanoMIPs-SPR-POF sensor incubated with increased concentrations of Cyt C (1 pM–10 nM). (**C**) Plasmonic spectra of GreenNanoMIPs-SPR-POF sensor incubated with increased concentrations of HSA (1 pM–10 nM). (**D**) Plasmonic resonance wavelength shifts (|Δ*λ*|) as a function of protein concentrations for cTnI (black square), Cyt C (red circles), and HSA (blue triangles). (**E**) Binding curve of NR10, showing the plasmonic resonance wavelength (*λ*) as a function of cTnI concentration, fitted with the Hill equation model (blue line).

The cTnI_nanoMIP-SPR sensor was further tested for its ability to detect the full-length cTnI in PBS, across a concentration range spanning from 100 fM to 10 nM. Figure 5A reports the plasmonic spectra obtained at different cTnI concentrations after 10 min of incubation using the same measurement protocol previously described. Zoomed-in views of the resonance peaks for cTnI, binding experiments are provided in the Supplementary Materials (Figure S4A). As observed, increasing cTnI concentrations induced a progressive blue-shift in the resonance wavelength, reaching a maximum |Δ*λ*| of approximately 2 nm. This shift indicates an alteration in the local refractive index at the sensor surface due to the specific interaction between the GreenNanoMIPs and the target protein, consistent with the behavior previously observed for the binding of the NR10 epitope peptide used as a template. In this case as well, the blue-shift can be attributed to the soft and hydrogel-like nature of the GreenNanoMIP layer, whose hydration-dependent swelling/shrinking and binding-induced deformation modify the optical properties of the plasmonic interface, resulting in a net shift toward shorter wavelengths.

To assess the selectivity of the cTnI_nanoMIP-SPR sensor, Cyt C and HSA were selected as non-target, competitive proteins. Both proteins were selected due to their high abundance in human plasma and their potential to interfere in complex biological matrices, especially in diagnostic applications, despite significantly differing from cTnI in terms of molecular weight, structure, and isoelectric point. Solutions of Cyt C and HSA tested over a concentration range from 1 pM to 10 pM did not induce any significant resonance wavelength shift (Figure 5B,C), indicating negligible non-specific binding and high selectivity of the cTnI_nanoMIP-SPR sensor (zoomed-in views of the shifts in Supplementary Information Figure S4B,C). As shown in the dose–response curves in Figure 5C, a clear binding response was observed exclusively for the analyte cTnI (black squares), while interfering proteins (Cyt C: red circles; HSA: blue triangles) did not produce significant signal variations. As previously described for the peptide binding experiments, the |Δ*λ*| values were used to construct the dose–response curves for data normalization across different chips, while the absolute resonance wavelengths (*λ*) were employed for Hill model fitting to extract the sensor parameters directly. Each measurement was repeated three times using independently fabricated chips, and the error bars represent the standard deviations across replicates. The experimental data for cTnI binding were fitted with the Hill equation model using OriginPro software (Figure 5E), and the corresponding extracted parameters are summarized in Table 3. The cTnI_nanoMIP-SPR sensor demonstrated exceptional sensitivity, with an LOQ of 1.17 × 10^−14^ M, a sensitivity at low detection of 8.49 × 10^13^, and an LOD of 3.53 × 10^−15^ M.

### 3.5. Measurement of cTnI in Serum

**Figure 6:**
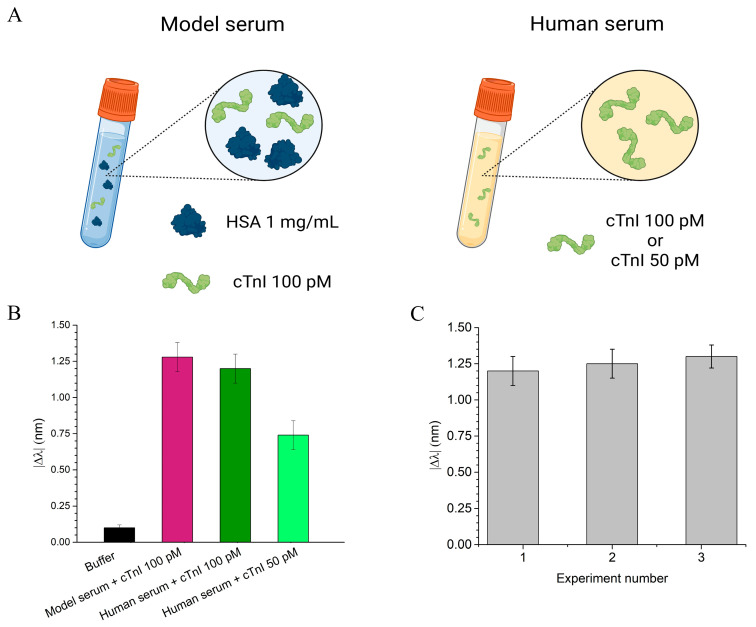
Evaluation of the cTnI_nanoMIP-SPR sensor performance in real samples. (**A**) Schematic representation of the tested samples: on the left, model serum consisting of HSA 1 mg/mL spiked with cTnI 100 pM; on the right, diluted human serum (1:50) spiked with cTnI at 100 pM or 50 pM. (**B**) Plasmonic resonance wavelength shifts (|Δ*λ*|) in different sample matrices: model serum spiked with 100 pM of cTnI (pink bar), and human serum spiked with 100 pM (dark green bar) and 50 pM (light green bar) of cTnI. (**C**) Reproducibility test: sensor response in three independent experiments performed with human serum spiked with 100 pM cTnI.

To assess the performance of the cTnI_nanoMIP-SPR sensor under biological conditions, we first tested the sensor using a model serum and then a real human serum from healthy individuals (provided by a commercial source, see Materials and Methods). The model serum was created by dissolving HSA at a concentration of 1 mg/mL. This concentration was chosen because HSA is the most abundant protein in human blood, typically present at around 35–50 mg/mL, and the 1 mg/mL concentration was used to simulate the protein composition found in diluted human serum (1:50), which is among the typical conditions used for serum measurements [20]. The commercial human serum samples were diluted 1:50 to ensure consistency in protein concentration across the testing conditions. Both the model and commercial diluted human serum thus represent complex biological matrices suitable for evaluating the sensor’s ability to detect cTnI in realistic conditions. The use of undiluted serum in plasmonic sensing is known to introduce substantial biofouling due to the high abundance of albumin, immunoglobulins, lipids, and other macromolecules that strongly adsorb onto the surfaces, which can interfere with the accessibility of the recognition layer and compromise the stability of the plasmonic signal. For this reason, the serum was diluted 1:50, a condition commonly adopted to mitigate matrix effects while preserving clinically relevant analyte levels. Notably, the clinical threshold associated with elevated cTnI (≥0.04 ng/mL) corresponds to 0.0008 ng/mL after a 1:50 dilution, still above the LOD of the present sensor (0.000084 ng/mL). Thus, the dilution strategy reduces nonspecific adsorption without compromising the sensor’s ability to detect cTnI at clinically meaningful concentrations. To further minimize non-specific interactions often caused by the complex serum matrix, Tween 20 at 0.1% *v*/*v* was added to the samples prior to incubation. This optimization effectively suppressed background signals originating from serum components, enhancing the sensor’s specificity and overall detection accuracy (Figure S5). Both model and real serum samples were spiked with defined concentrations of cTnI: 100 pM for the model serum, and 50 pM and 100 pM for the human serum. As illustrated in Figure 6B, the model serum spiked with 100 pM cTnI produced a resonance wavelength shift of approximately 1.3 ± 0.1 nm (pink bar). Similarly, in the diluted human serum, the sensor yielded a shift of 1.29 ± 0.1 nm for 100 pM cTnI (dark green bar) and a distinct but lower shift of 0.7 nm for 50 pM cTnI (light green bar). Notably, the ability to detect cTnI well below the clinical threshold (160 pM) highlights the potential of the cTnI_nanoMIP-SPR sensor for early-stage diagnosis of myocardial injury. The reproducibility of the sensor was evaluated by testing multiple cTnI_nanoMIP-SPR sensors across different experimental runs. For each set of measurements, at least three separate sensor chips were used, and experiments were conducted on different days to ensure consistency over time. The response to a 100 pM cTnI solution was measured for each chip (*n*= 3), with the resulting resonance shift recorded as 1.25 ± 0.05 nm, as shown in Figure 6C, confirming good reproducibility of the sensor platform.

## 4. Discussion

In this work, the coupling of GreenNanoMIPs with a portable D-shaped POF-SPR transducer resulted in the development of an optical sensor capable of detecting the myocardial infarction marker cTnI in serum samples, within the critical clinical range [2]. The performance of the GreenNanoMIP-SPR sensor was evaluated through a series of binding experiments, demonstrating its ability to detect both the NR10 peptide epitope and the full-length cTnI marker within minutes, with high sensitivity and selectivity. The sensor’s applicability under clinically relevant conditions was further validated by testing its performance in real serum samples. The integration of biomimetic GreenNanoMIPs with a portable D-shaped POF-SPR platform led to a sensor with an LOD of 3.53 × 10^−15^ M. This value is significantly lower than those reported for previously developed sensor platforms, positioning it as a promising tool for the early detection of myocardial infarction. For comparison, a list of sensors for cTnI are reported in Table 4.

Choudhary and Altintas et al. [20] reported an SPR-based sensor for cTnI detection, which displayed an LOD of 2.16 × 10^−9^ M. The SPR sensor was based on a conventional Kretschmann-configuration setup and made use of nanoMIPs with an average hydrodynamic size of 150 nm and a PDI of 0.150, prepared from a combination of acrylamides and *N*-isopropylacrylamide. The LOD achieved was remarkably similar to the results achieved for the detection of cTnI with SPR sensors equipped with monoclonal antibodies anti-cTnI [40,41]. In contrast, when the MIP was in the form of a thin layer of a few nanometers, prepared by a controlled electropolymerization of norepinephrine (NE), used as the functional monomer, and layered onto the gold chip of a Kretschmann-configuration SPR (i.e., Biacore), the sensor LOD was in the range of hundreds of pM. In SPR, binding events are detected as a change in local refractive index at the interface between the metal and the dielectric. It is expected that the ultrathin layer of poly-NE, deposited directly to the sensing interface, improves the transduction of the cTnI binding, as the specific recognition cavities on the poly-NE layer are within a distance of a few nanometers from the plasmon wave [42]. Finally, the herein reported SPR sensor based on castor oil-derived nanoMIPs was shown to exhibit a femtomolar LOD, surpassing both antibody-based SPR and nanoMIPs Kretschmann SPR approaches, while achieving an LOD similar to poly-NE MIP layers. Among the key factors contributing to this performance, there are both the herein-used multimode POF SPR setup and the characteristics of the GreenNanoMIP colloidal suspension. The multimode POF SPR setup, tested with solutions of known RI, was estimated to have a sensitivity value (S_n_) of 1308 × 10^3^ (nm/RIU) and a figure of merit (FOM) of 13.34 RIU^−1^ for pure water (*n* = 1.332) [44]. Concerning the GreenNanoMIP nanoparticles used as selective receptors, these display a strikingly high uniformity, as shown in Table S1. The GreenNanoMIPs exhibited a PDI equal to 0.064, which is remarkably low and indicates a very consistent particle size. It is expected that such receptors’ uniformity translates into an extremely homogeneous recognition layer and into accessible binding sites, facilitating an efficient target recognition and high reproducibility of the sensor responses, as discussed earlier [45]. Additionally, the GreenNanoMIPs hydrodynamic diameter is around 80 nm, which is half the size of the cTnI nanoMIPs reported earlier in an SPR sensor [20]. As in SPR, the plasmon interrogating wave is governed by an exponential decay from the metallic surface towards the dielectric space, keeping the binding event within a distance of 80 nm from the metallic surface; with respect to monitoring binding events at 150 nm from the metal surface, these can be calculated to improve the sensitivity of the measure of a factor of about 20–44 times, depending on the interrogating wavelength. At last, the GreenNanoMIPs are not only derived from a renewable source, but also exhibited an outstanding stability (up to one year in solution), supporting the solidity of the sensor’s response. Beyond MIP- and antibody-based SPR platforms, Table 4 also includes representative immunoassay technologies commonly employed for cTnI quantification, such as enzyme-linked immunosorbent assays (ELISAs) [4,43] and chemiluminescent immunoassays (CLIAs) [5]. These methods typically provide dynamic ranges spanning from the low-nanogram per mL region up to hundreds of nanograms per mL, with an LOD extending from the sub-nanogram per mL level for ELISAs down to the sub-picogram per mL in the case of CLIAs. Such values reflect the sensitivity currently achievable in clinical diagnostics and define the benchmarks against which emerging biosensing approaches must be evaluated. When considered within this broader analytical landscape, the femtomolar LOD achieved by the cTnI_nanoMIP-SPR sensor falls well within, and in some cases exceeds, the sensitivity range characteristic of state-of-the-art immunoassay methodologies.

Although the present study demonstrated the feasibility of cTnI detection in diluted serum samples, the use of undiluted serum would be expected to introduce more pronounced biofouling effects due to the high content of albumin, immunoglobulins, lipids, and other macromolecules that can strongly adsorb onto plasmonic metal surfaces. Such nonspecific deposition may progressively mask the recognition layer and reduce the stability of the sensing interface during repeated measurements. While these aspects were beyond the scope of this work, future developments could integrate antifouling strategies to enhance robustness against cumulative fouling and to support measurements in more complex or minimally processed clinical samples. Overall, the present results provide the basis for an optical-sensor solution translatable into a tool for early diagnosis, offering a promising alternative in response to clinical needs.

## Figures and Tables

**Table 1:** Chemical characterization determined by XPS of the different steps of chip functionalization, as shown in Figure 1. XPS data are acquired at 0° take-off angle, and the standard error does not exceed 1–2% of the reported value.

	C (%)	O (%)	N (%)
Bare	9.40	6.02	/
SAM	46.30	25.63	/
GreenNanoMIP	56.00	23.50	4.70

**Table 2:** Fitting parameters and sensor parameters related to GreenNanoMIPs-SPR-POF sensor’s response to NR10.

Parameters	Value	
*λ__start_* (nm)	613.45 ± 0.22	
*λ*__*end*_ (nm)	610.63 ± 0.18	
*K_app_* (M)	2.73 × 10^−11^ ± 1.38 × 10^−11^	
*n*	0.49 ± 0.12	
R^2^_adj_	0.96589	
Χ^2^_red_ **	0.90761	
K_aff_ (M^−1^)	3.66 × 10^10^	K_aff_ = 1/*K_app_*
Sensitivity at low concentration (nm/M)	2.23 × 10^13^	|*λ__end_* − *λ__start_*|/*K_app_*
LOQ (M)	9.83 × 10^−14^	10 × St.Dev. _blank_/Sensitivity _low conc_ *
LOD (M)	2.9 × 10^−14^	3 × St.Dev. _blank_/Sensitivity _low conc_ *

* St.Dev. _blank_ represents the standard deviation measured when buffer is added to the sensor. ** Statistical indicator of the goodness of the fit.

**Table 3:** Fitting parameters and sensor parameters related to GreenNanoMIPs-SPR-POF sensor’s response to cTnI.

Parameters	Value	
*λ__start_* (nm)	608.29 ± 0.10	
*λ__end_* (nm)	606.49 ± 0.05	
*K_app_* (M)	7.14 × 10^−12^ ± 1.45 × 10^−12^	
*n*	1.11 ± 0.20	
R^2^_adj_	0.98266	
Χ^2^_red_ **	0.56951	
K_aff_ (M^−1^)	1.40 × 10^11^	K_aff_ = 1/*K_app_*
Sensitivity at low concentration (nm/M)	8.49 × 10^13^	|*λ__end_* − *λ__star_*_t_|/*K_app_*
LOQ (M)	1.17 × 10^−14^	10 × St.Dev. _blank_/Sensitivity _low conc_ *
LOD (M)	3.53 × 10^−15^	3 × St.Dev. _blank_/Sensitivity _low conc_ *

* St.Dev. _blank_ represents the standard deviation measured when buffer is added to the sensor. ** Statistical indicators of the goodness of the fit.

**Table 4:** List of SPR direct biosensors used for the detection of cTnI biomarkers reported in the literature and the current work.

Receptor Type	Detection	Range Concentration	LOD	Reference
Anti-cTnI antibody on fiber optic SPR	SPR	2500–4000 ng/mL	1 ng/mL	[40]
Monoclonal-anti-cTnI antibody on SPR	SPR	0.5–20 ng/mL	0.25 ng/mL	[41]
NanoMIPs grafted to Au on Kretschmann SPR	SPR	0.78–50 ng/mL	0.52 ng/mL	[20]
Norepinephryne MIP layer electropolymerized on Au chip for Biacore SPR	SPR	2.5–20 nM i.e., 6–48 ng/mL	460 pM i.e., 0.000011 ng/mL	[42]
GreenNanoMIPs grafted to Au on D-shaped POF-SPR	SPR	1 pM–100 nM i.e., 0.024 to 240 ng/mL	0.000084 ng/mL	This work
ELISA		0.32–200 ng/mL	0.32 ng/mL	[4]
ELISA HRP-based fluorescent		5–180 ng/mL	0.19 ng/mL	[43]
CLIA		Not indicated	0.116 pg/mL	[5]

## Data Availability

Data are available upon request.

## Supplementary Materials

The following supporting information can be downloaded at https://www.mdpi.com/article/10.3390/bios16010012/s1, “Supplementary Material Marinangeli et al.” contains: Table S1: Particle size and the PDI of GreenNanoMIPs; Figure S1: Particle size distributions of GreenNanoMIPs at the initial measurement and subsequently after one year. Re-printed from [22]; Figure S2: Deconvolution analysis of high-resolution XPS spectra of bare Au SPR-chip, SAM-modified SPR-chip and GreenNanoMIP functionalized SPR-chips; Figure S3: Zoomed-in view of the plasmonic spectra of GreenNanoMIPs-SPR-POF sensor incubated with increased concentrations of NR10 peptide (A) and with increased concentrations of NR11 peptide (B); Figure S4: Zoomed-in view of the plasmonic spectra of GreenNanoMIPs-SPR-POF sensor incubated with increased concentrations of cTnI (A), HSA (B) and Cyt C (C); Figure S5: Evaluation of the effect of the addition of 0.1% of Tween20 to the working buffer on the cTnI_nanoMIP-SPR-sensor performance when tested with real samples. In the absence of Tween20, human serum 1:50 (red bar) and human serum 1:50 spiked with 100 pM of cTnI (blue bar) showed similar effects on the sensor (*λ* = 624.16 nm and *λ* = 624.53 nm), demonstrating that non-specific absorption occurring at the sensor’s surface was the prevailing mechanism. In contrast, when Tween20 was added to the buffer, the matrix effect on the sensor observed for diluted human serum (red bar) accounted for a *λ* = 614.55 nm, indicating a reduced matrix effect. More importantly, when human serum 1:50 spiked with 100 pM of cTnI (blue bar) was placed on the sensor, the expected blue-shift (*λ* = 612.84 nm) that correlated with the cTnI spiked concentration was observed; Figure S6: SEM image of GreenNanoMIPs.

## Abbreviations

The following abbreviations are used in this manuscript:

MIPs	Molecularly imprinted polymers
cTnI	Cardiac troponin I
POF	Plastic optical fiber
SPR	Surface plasmon resonance
