# Supplementary material for: Highly Sensitive Biosensor for the Detection of Cardiac Troponin I in Serum via Surface Plasmon Resonance on Polymeric Optical Fiber Functionalized with Castor Oil-Derived Molecularly Imprinted Nanoparticles
Source: Biosensors (Basel). 2025 Dec 23;16(1):12. doi: 10.3390/bios16010012 (PMC12839181; doi:10.3390/bios16010012)
Supplement: Supplementary file 1 [file biosensors-16-00012-s001.zip › biosensors-4001210-supplementary.pdf]

biosensors

MDPI

*Supplementary Materials*

# Highly Sensitive Biosensor for the Detection of Cardiac Troponin I in Serum via Surface Plasmon Resonance on Polymeric Optical Fiber Functionalized with Castor Oil-Derived Molecularly Imprinted Nanoparticles

**Alice Marinangeli [1,*], Pinar Cakir Hatir [2,3], Mustafa Baris Yagci [4] and Alessandra Maria Bossi [1,*]**

[1] Department of Biotechnology, University of Verona, Strada Le Grazie 15, 37134 Verona, Italy
[2] Department of Biomedical Engineering, Faculty of Engineering and Natural Sciences, İstinye University, Ayazağa Mah. Azerbaycan Cad. (Vadistanbul 4A Blok) Sariyer, İstanbul 34396, Türkiye; pinar.hatir@istinye.edu.tr
[3] Nanotechnology and Advanced Materials Research Center, Istinye University, Sariyer, Istanbul 34396, Türkiye
[4] Koç University Surface Science and Technology Center (KUYTAM), Istanbul 34450, Türkiye; byagci@ku.edu.tr
* Correspondence: alice.marinangeli@univr.it (A.M.); alessandramaria.bossi@univr.it (A.M.B.); Tel.: +39-045-8027-833 (A.M.); Tel.: +39-045-8027-946 (A.M.B.)

**1. Hydrodynamic size and polydispersity index (PDI) of GreenNano-MIPs**

**2. Stability of GreenNanoMIP over time**

**3. X-Ray Photoelectron Spectroscopy**

**4. Zoomed-in view of the plasmonic spectra of GreenNanoMIPs-SPR-POF for the binding of NR10 and NR11 peptide**

**5. Zoomed-in view of the plasmonic spectra of GreenNanoMIPs-SPR-POF for the binding of cTnI, HSA and Cyt C**

**6. Measurement of cTnI in serum with and without Tween20**

**7. SEM imaging of GreenNanoMIPs**

 https://doi.org/10.3390/bios16010012

## 1. Hydrodynamic size and polydispersity index (PI) of GreenNanoMIPs

**Table S1.** Particle size and the PDI of GreenNanoMIPs.

| Sample | Particle size (nm) | PDI |
|---|---|---|
| GreenNanoMIPs | 80.9 | 0.064 |

## 2. Stability of GreenNanoMIP over time

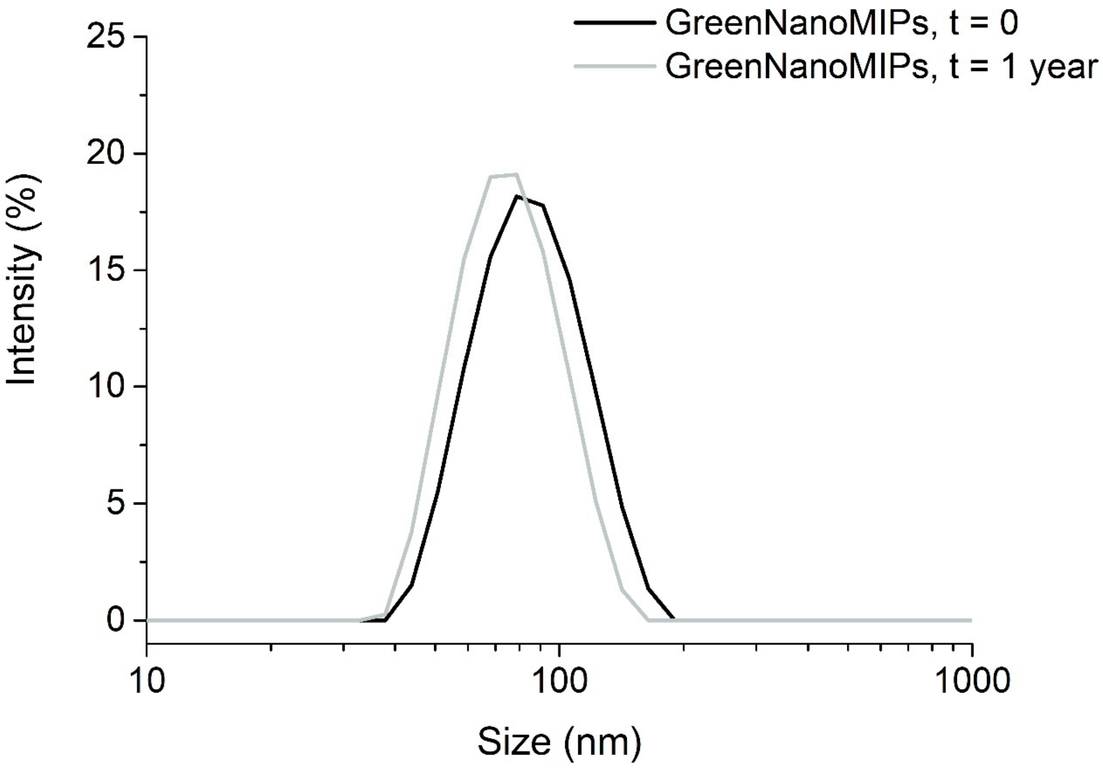


**Figure S1.** Particle size distributions of GreenNanoMIPs at the initial measurement and subsequently after one year. Re-printed from [22].

## 3. X-Ray Photoelectron Spectroscopy

https://doi.org/10.3390/bios16010012

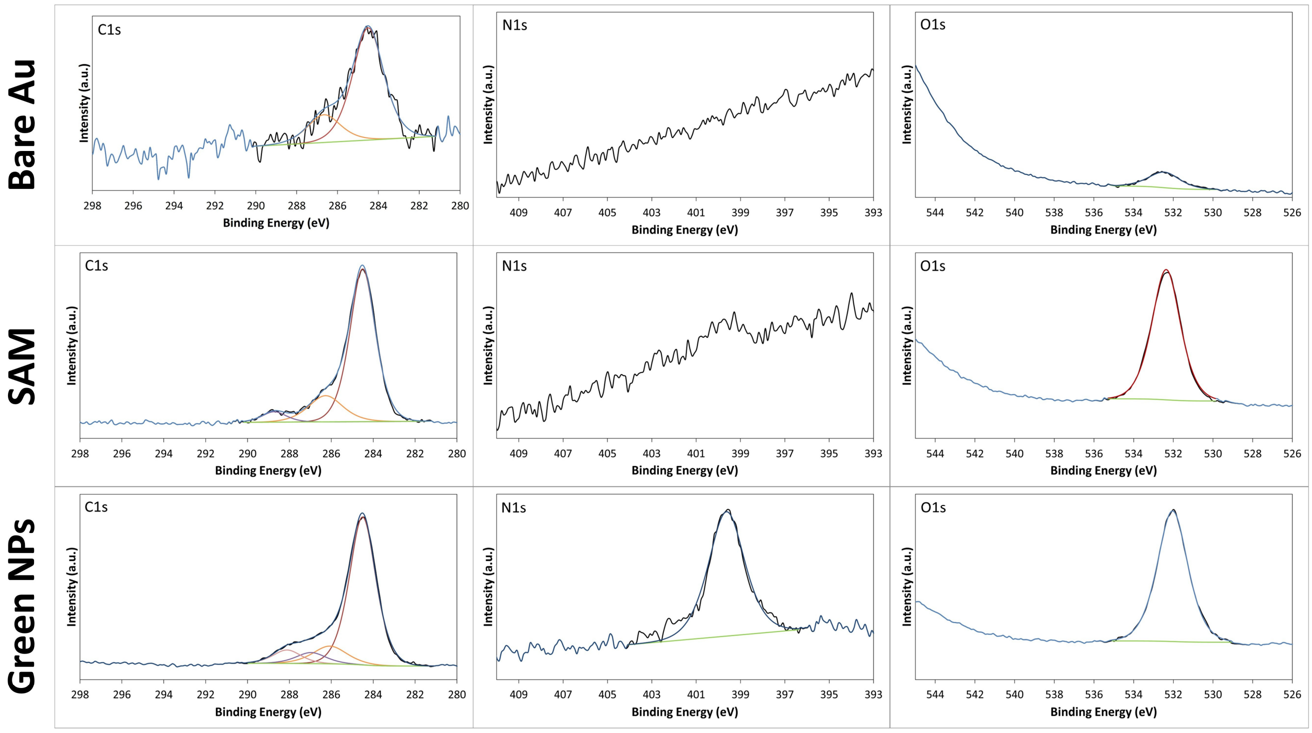


**Figure S2.** Deconvolution analysis of high-resolution XPS spectra of bare Au SPR-chip, SAM-modified SPR-chip and GreenNanoMIP functionalized SPR-chips.

## 4. Zoomed-in view of the plasmonic spectra of GreenNanoMIPs-SPR-POF for the binding of NR10 and NR11 peptide

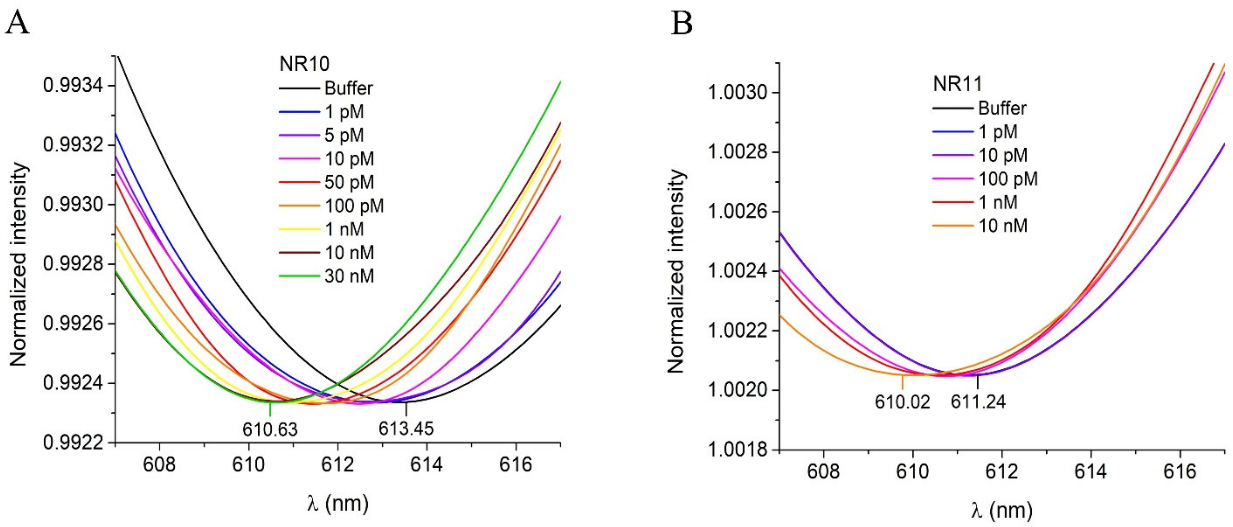


**Figure S3.** Zoomed-in view of the plasmonic spectra of GreenNanoMIPs-SPR-POF sensor incubated with increased concentrations of NR10 peptide (A) and with increased concentrations of NR11 peptide (B).

## 5. Zoomed-in view of the plasmonic spectra of GreenNanoMIPs-SPR-POF for the binding of cTnI, HSA and Cyt C

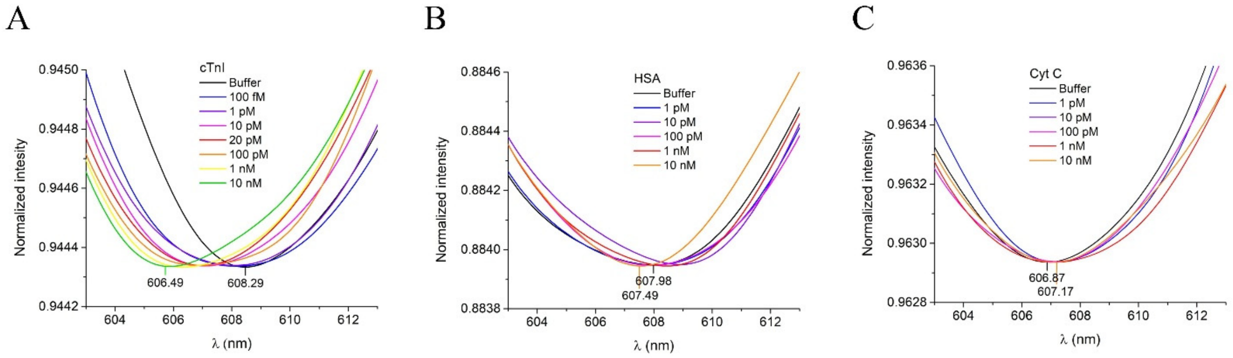


**Figure S4.** Zoomed-in view of the plasmonic spectra of GreenNanoMIPs-SPR-POF sensor incubated with increased concentrations of cTnI (A), HSA (B) and Cyt C (C).

## 6. Measurement of cTnI in serum with and without Tween20

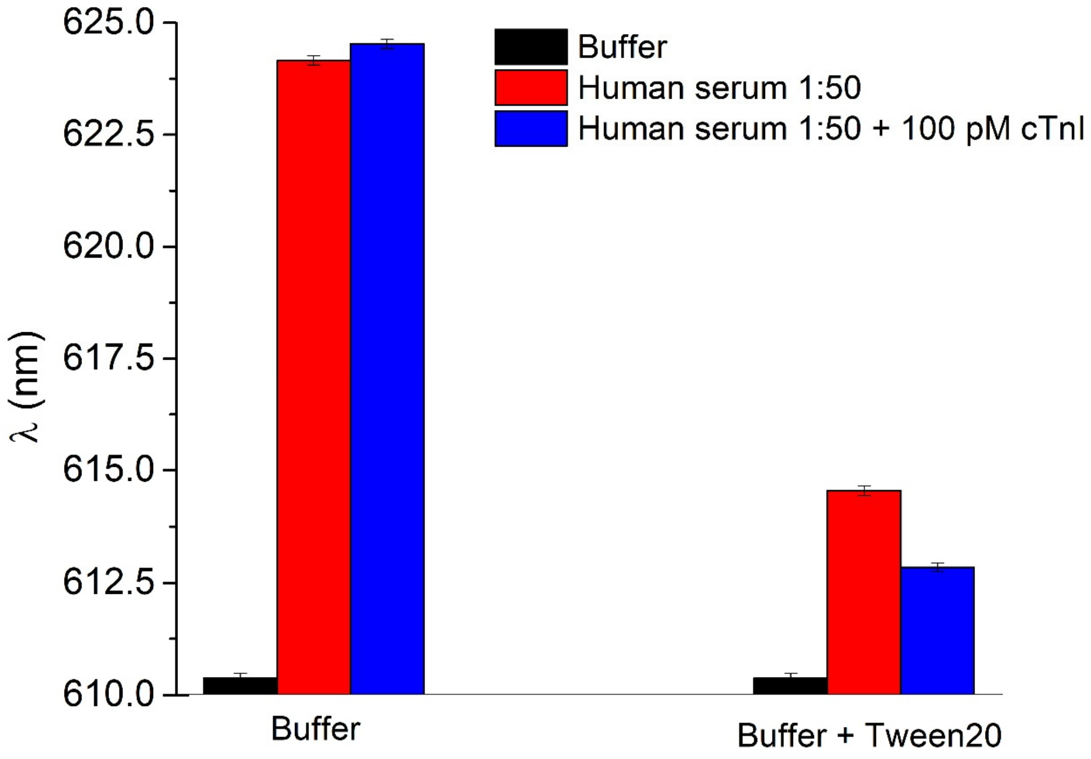


**Figure S5.** Evaluation of the effect of the addition of 0.1% of Tween20 to the working buffer on the cTnI_nanoMIP-SPR-sensor performance when tested with real samples. In the absence of Tween20, human serum 1:50 (red bar) and human serum 1:50 spiked with 100 pM of cTnI (blue bar) showed similar effects on the sensor ($\lambda$= 624.16 nm and $\lambda$= 624.53 nm), demonstrating that non-specific absorption occurring at the sensor's surface was the prevailing mechanism. In contrast, when Tween20 was added to the buffer, the matrix effect on the sensor observed for diluted human serum (red bar) accounted for a $\lambda$ = 614.55 nm, indicating a reduced matrix effect. More importantly, when human serum 1:50 spiked with 100 pM of cTnI (blue bar) was placed on the sensor, the expected blue-shift ($\lambda$= 612.84 nm) that correlated with the cTnI spiked concentration was observed.

## 7. SEM imaging of GreenNanoMIPs

SEM images were collected with a JCM-5000 NeoScope QUATTRO S. Prior to SEM analysis, the GreenNanoMIPs were resuspended in distilled water to a final concentration

of 1 mg/mL. A small volume of the sample was drop-casted onto a clean surface, air-dried and subsequently examined under SEM. Figure S6 shows the GreenNanoMIPs at different magnifications. The size of the GreenNanoMIPs matches with DLS data.

It is worth noting that the air-drying process of GreenNanoMIPs yields aggregation artefacts.

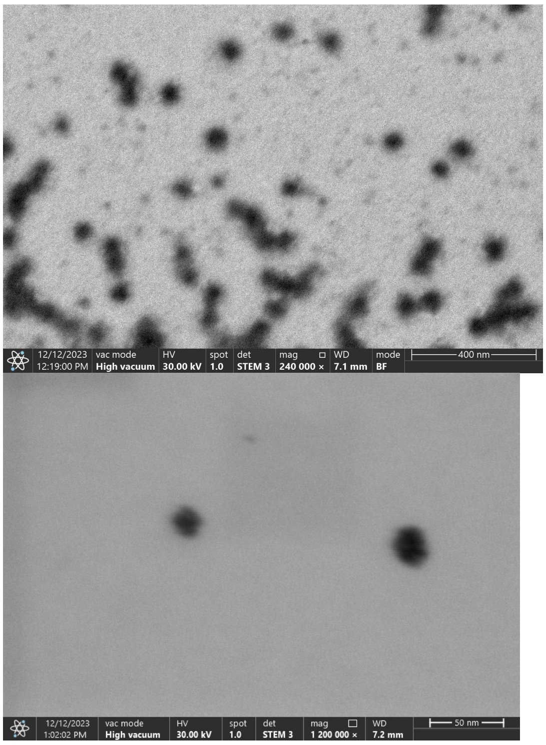


**Figure S6.** SEM image of GreenNanoMIPs.

https://doi.org/10.3390/bios16010012
